# Assembly methods for nanopore-based metagenomic sequencing: a comparative study

**DOI:** 10.1038/s41598-020-70491-3

**Published:** 2020-08-12

**Authors:** Adriel Latorre-Pérez, Pascual Villalba-Bermell, Javier Pascual, Cristina Vilanova

**Affiliations:** Darwin Bioprospecting Excellence S.L., Paterna, Spain

**Keywords:** Bioinformatics, Sequencing, Microbial communities, Microbial genetics

## Abstract

Metagenomic sequencing has allowed for the recovery of previously unexplored microbial genomes. Whereas short-read sequencing platforms often result in highly fragmented metagenomes, nanopore-based sequencers could lead to more contiguous assemblies due to their potential to generate long reads. Nevertheless, there is a lack of updated and systematic studies evaluating the performance of different assembly tools on nanopore data. In this study, we have benchmarked the ability of different assemblers to reconstruct two different commercially-available mock communities that have been sequenced using Oxford Nanopore Technologies platforms. Among the tested tools, only metaFlye, Raven, and Canu performed well in all the datasets. These tools retrieved highly contiguous genomes (or even complete genomes) directly from the metagenomic data. Despite the intrinsic high error of nanopore sequencing, final assemblies reached high accuracy (~ 99.5 to 99.8% of consensus accuracy). Polishing strategies demonstrated to be necessary for reducing the number of indels, and this had an impact on the prediction of biosynthetic gene clusters. Correction with high quality short reads did not always result in higher quality draft assemblies. Overall, nanopore metagenomic sequencing data-adapted to MinION’s current output-proved sufficient for assembling and characterizing low-complexity microbial communities.

## Background

Metagenomic sequencing has revolutionized the way we study and characterize microbial communities. This culture-independent technique based on shotgun sequencing has been applied in a broad range of biological fields, ranging from microbial ecology^1^ to evolution^2^, or even clinical microbiology^3^. In recent years, metagenomics has also become a powerful tool for recovering individual genomes directly from complex microbiomes^2,4,5^, leading to the identification and description of new- and mostly unculturable-taxa with meaningful implications^6^.

Illumina has been the most widely used platform for metagenomic studies. Illumina reads are characterized by their short length (75–300 bp) and high accuracy (~ 0.1% of basecalling errors)^7^. When performing de novo assemblies, Illumina sequences often result in highly fragmented genomes, even when sequencing pure cultures^8,9^. This is a consequence of the inability to correctly assemble genomic regions containing repetitive elements that are longer than the read length^9^. This fragmentation problem is magnified when handling metagenomic sequences due to the existence of intergenomic repeats that are shared by more than one taxon present in the microbial community^10^. It has to be noted that microbial communities often contain related species or sub-species in different-and unknown- abundances, resulting in extensive intergenomic overlaps that can hinder the assembly process^11,12^.

Third generation sequencing platforms have recently emerged as a solution to resolve ambiguous repetitive regions and to improve genome contiguity. Despite the considerable error associated to these technologies (~ 5 to 15% of basecalling errors)^13,14^, their ability to produce long reads (up to 10–12 kb of mean read length)^7,15^ has allowed them to generate genomes with a high degree of completeness^16,17^. Currently, the most widely-used third generation technologies are Pacific Biosciences (PacBio) and Oxford Nanopore Technologies (ONT), both based on single molecule sequencing, and therefore, PCR-free. PacBio was the first long-read technology established in the market^18^. However, PacBio instruments require particular operation conditions and large capital investments^19^. On the other hand, ONT platforms are becoming increasingly popular among researchers, especially in the case of MinION sequencers. Although the cost of GridION and PromethION devices is also notable (~ 50,000$ to 170,000$), MinION is a cost-effective (~ 1,000$), portable sequencing platform, which enables real-time analysis pipelines^20^. This platform has been broadly applied over the last few years, due to its suitability for in-field and clinical studies^21,22^, but also for sequencing complete prokaryotic and eukaryotic genomes^17,23–25^, and characterizing microbial communities^26,27^.

Benchmarking is the usual way to evaluate genomic methodologies (i.e. DNA extraction, library preparations, etc.) and bioinformatic tools. In the metagenomic context, benchmarking studies are frequently based on mock communities. A mock community is an artificial microbial community in which the abundance of all the microorganisms is known^28^. Mock communities can be generated in silico^29^ or experimentally, as a mixture of defined DNA proportions. For de novo assemblies, a great effort has been made in order to benchmark all the available tools and methodologies suitable for studying microbial ecosystems via Illumina shotgun sequencing^12,30,31^. Nevertheless, due to the highly dynamic development of new software applicable to ONT platforms, we found that the few evaluation studies that have been focused to date on nanopore-based metagenomic assembly did not cover the current spectrum of available assemblers^32–34^.

In the present study, we have used the data generated by Nicholls et al.^15^ to comprehensively assess the current state-of-art of *de novo* assembly tools suitable for nanopore-based, metagenomic sequencing. Original data was generated through metagenomic sequencing of two microbial communites (ZymoBIOMICS Microbial Community Standards CS and CSII) with both GridION and PromethION platforms. Overall, this work demonstrates the suitability of using nanopore sequencing exclusively for assembling low-complexity microbial communities, and paves the way towards the standardization of bioinformatic pipelines for long-read sequencing data.

## Methods

### Dataset description

Benchmarking datasets were extracted from Nicholls et al.^15^ (PRJEB29504), and consisted of high-coverage sequencing of two individual mock communities (ZymoBIOMICS Microbial Community Standards CS Even ZRC190633 and CSII Log ZRC190842) with both GridION and PromethION platforms. The mock communities contained the same species (eight bacteria; two yeasts), but differed in the expected proportion for each microorganism. CS mock community has a homogeneous distribution of microorganisms (12% for each bacteria and 2% for the yeasts), while the species present in CSII are distributed on a logarithmic scale, with relative abundances ranging from 89.1 to 0.000089% (Table 1). Following the nomenclature from Nicholls et al.^15^, we have used the terms “Even” when referring to CS mock community, and “Log” when referring to CSII.

**Table 1:** Description of the microorganisms comprising the ZymoBIOMICS mock communities and their theoretical composition.

Species	Abbreviations	Estimated size (Mbp)	Composition even (CS) (%)	Composition log (CSII) (%)
*Bacillus subtilis*	*B. subtilis*	4.134	12.00	0.89
*Cryptococcus neoformans*	*C. neoformans*	18.599	2.00	0.00089
*Enterococcus faecalis*	*En. faecalis*	2.965	12.00	0.00089
*Escherichia coli*	*E. coli*	5.140	12.00	0.089
*Lactobacillus fermentum*	*L. fermentum*	2.012	12.00	0.0089
*Listeria monocytogenes*	*Li. monocytogenes*	3.008	12.00	89.1
*Pseudomonas aeruginosa*	*P. aeruginosa*	6.592	12.00	8.9
*Saccharomyces cerevisiae*	*S. cerevisiae*	11.864	2.00	0.89
*Salmonella enterica*	*Sa. enterica*	4.781	12.00	0.089
*Staphylococcus aureus*	*St. aureus*	2.838	12.00	0.000089

Nicholls et al.^15^ yielded  ~ 14 Gbp of data on a single GridION flowcell (48 h of sequencing) and ~ 152 Gbp on the PromethION platform (64 h of sequencing). In order to reduce the computational effort, we performed an initial subsampling of this data. In particular, GridION and PromethION datasets were subsampled at two different sequencing depths (3 Gbp and 6 Gbp) to recreate MinION runs with different outputs, and the yield matched the output described in recent shotgun sequencing experiments based on MinION^9,34–39^. Subsampling was performed by selecting the top lines of the FASTQ files. Nevertheless, the most promising tools were further tested on the original GridION data to check their computational demands and general performance. All the datasets were trimmed with porechop (https://github.com/rrwick/Porechop; v. 0.2.4) in order to remove adapters from read ends and split sequences with internal adapters.

### De novo assembly

As first proposed by Lindgreen et al.^40^, the tools selected for the present benchmarking were required to meet the following criteria:

- The tool should be freely available.
- The tool should have a suitable user guide, both for installation and usage.
- The tool should have been extensively used or show potential to become widely used.

In our study, a total of three widely used metagenomic short-read assemblers and ten long-read tools (or different versions of the same tool) were taken into consideration. Nevertheless, it was not possible to install and/or run all the software due to different reasons (Supplementary Table S1). The commands used for running each assembler are provided in Supplementary Table S2. It is worth highlighting that tools were run with default parameters when no metagenomic configuration was explicitly recommended in the user guide.

### Reference genomes

All the species included in the mock community had an available reference genome sequenced with a combination of Illumina and nanopore reads (available at https://doi.org/10.5281/zenodo.3935737). These assemblies provided by Zymo Research Corporation (Irvine, CA, USA) consisted of eight complete genomes for the bacterial strains, and two draft genomes for the yeasts.

Nicholls et al.^15^ sequenced and assembled each genome again from pure cultures using Illumina reads only. In the present work, however, ZymoBIOMICS genomes were used as a reference for carrying out the comparative analyses, due to their higher level of completeness. Although these reference genomes cannot be considered as “gold standards”, Goldstein et al.^9^ demonstrated that the error profile obtained through hybrid assembly (ONT + Illumina MiSeq) was similar to the one obtained with MiSeq-only assembly, but the former resulted in higher contiguity. Reference genomes were gathered in a single multi-FASTA file to create a single-reference metagenome.

### Evaluation of the assemblies

All the assemblers were run on the same desktop computer (CPU: AMD RYZEN 7 1700X 3.4GHZ; Cores: 8; Threads: 16; RAM: Corsair Vengeance 64 GB; SSD: Samsung 860 EVO Basic SSD 500 GB) working under Ubuntu 18.04 operative system. The time required by each tool to perform the assembly was measured with the built-in bash version of the “time” command.

Completeness and contiguity of de novo assemblies were first evaluated via QUAST (v. 5.0.2)^41^. MetaQUAST (v. 5.0.2)^42^ was used for obtaining assembly statistics based on the alignment of the generated contigs against the reference genomes. Only contigs longer than 500 bp and with > X10 coverage were selected for calculating the general statistics. MetaQUAST failed to run with some draft metagenomes and, for that reason, minimap2 (v. 2.15)^43^ was used instead to align the assemblies to the reference metagenome. Then, the percentage of metagenome covered by the draft assemblies was calculated using the ‘pileup.sh’ script from BBTools suite (http://sourceforge.net/projects/bbmap/).

The resulting assemblies were further evaluated in order to determine their error profile. Due to the lack of a standard methodology, the presence of SNPs and indels was analyzed using two different strategies. The first one consisted of the alignment of the contigs against the reference metagenome via minimap2. BAM files were then analysed using bcftools (https://samtools.github.io/bcftools/; v. 1.9) and the in-house script ‘indels_and_snps.py’ (https://doi.org/10.5281/zenodo.3935763) was applied to quantify the variants. The second strategy was based on the use of MuMmer4 (https://sourceforge.net/projects/mummer/files/; v. 3.23). This tool was employed to align the draft assemblies to the reference metagenome. Then, the script ‘count_SNPS_indels.pl’ from Goldstein et al.^9^ was used to calculate the final number of SNPs and indels. In both strategies, the number of variants was normalized to the total assembly size of each metagenome.

Biosynthetic gene clusters (BGCs) are usually composed of repetitive genetic structures that are hard to assemble with short reads, and with long-read technologies being therefore more suitable to overcome this issue. However, BGCs are also very sensitive to frameshift errors, which have been reported to frequently occur in nanopore data^9^. For that reason, AntiSMASH web service (v. 5.0)^44^ was used to compare the performance on BGC prediction BGCs number and profile among the different assembly tools.

### Assembly polishing

Draft assemblies (Even GridION 6 Gbp dataset) were further polished with Racon ^45^ and Medaka (https://nanoporetech.github.io/medaka/), using the commands specified in Supplementary Table S2. As the Medaka model for the specific version of Guppy (v2.2.2 GPU basecaller) originally used for basecalling the data was not available, we used the Medaka default model (r941_min_high_g351) for polishing. ONT or Illumina reads were used for iteratively running 4 rounds of Racon. Polishing was carried out using the same ONT input reads as those used for assembling each dataset, whereas Illumina reads (MiSeq plataform) were retrieved from the shotgun metagenomic sequencing data available for the Even mock community (ERR2984773)^15^. Only the draft assemblies corrected with ONT reads were further polished with Medaka, again using the original ONT sequences as input. Indels and SNPs were evaluated after each polishing step using the MumMer-based strategy, as detailed above.

## Results

### Subsampling

In the present study, the data released by Nicholls et al.^15^ (ultra-deep sequencing of two different mock communities using GridION and PromethION platforms) was used in order to study the suitability of nanopore sequencing to characterize low complex microbial communities. The mock communities were composed by the same ten microorganisms, but in different proportions (Table 1). With the aim of reducing the computational resources needed for the first screening of the selected assemblers, the GridION and PromethION datasets were subsampled to obtain an output comparable with recent genomic or metagenomic studies based on MinION (approximately 3 Gbp and 6 Gbp)^9,34–39^. In general, mean read length remained the same in the subsampled datasets in comparison to the original sequencing data^15^. However, read quality was higher in the subsampled dataset. This fact suggested a bias towards higher qualities at the start of the run, since subsampling was carried out by selecting the top reads of the original files (Table 2). In fact, the bottom reads which are acquired later in the sequencing run displayed the same quality than the whole dataset.

**Table 2:** Sequencing statistics for the original and the subsampled datasets.

Dataset name	Original dataset				New dataset				
	Gbp	Number of reads	Mean read length	Mean read quality	Gbp	Number of reads	Mean read length	Mean read quality	SRA accession number
Even GridION	14.007	3,491,078.0	4,012.3	8.4	3.042	747,682.0	4,069.5	8.9	SRX6817349
Log GridION	16.032	3,667,007.0	4,372.0	8.0	3.053	685,926.0	4,451.0	8.7	SRX6817351
Even PromethION	146.291	36,527,376.0	4,005.0	7.3	2.979	748,367.0	3,981.0	8.2	SRX6817353
Log PromethION	148.028	35,118,078.0	4,215.2	7.6	2.990	711,524.0	4,203.3	8.3	SRX6817355
Even GridION	14.007	3,491,078.0	4,012.3	8.4	6.092	1,495,377.0	4,073.9	8.8	SRX6817350
Log GridION	16.032	3,667,007.0	4,372.0	8.0	6.094	1,371,820.0	4,442.4	8.5	SRX6817352
Even PromethION	146.291	36,527,376.0	4,005.0	7.3	5.970	1,496,919.0	3,988.8	8.2	SRX6817354
Log PromethION	148.028	35,118,078.0	4,215.2	7.6	5.956	1,422,918.0	4,185.8	8.2	SRX6817356

### Metagenome assembly

From the selected tools, we were able to correctly install and run nine out of the ten long-read assemblers, and two out of the three short-read assemblers (Supplementary Table S1). In total, 74 assemblies were generated, 40 for the Even mock community and 34 for the Log community. Six assemblies could not be completed because miniasm and Pomoxis failed to run with the 6 Gbp Log datasets, whereas Unicycler failed to run with the 3 Gbp Log datasets. The total size of each draft assembly and the fraction of metagenome recovered from the reference genomes were evaluated for the Even datasets in order to obtain a first view of the general tool performance.

**Figure 1:**
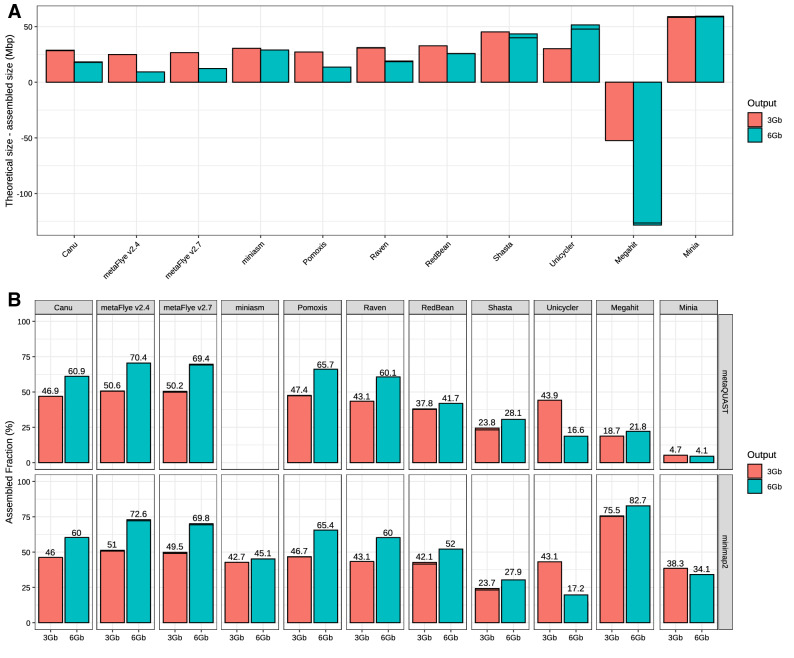
Evaluation of metagenome assembly size corresponding to each tested tool for the subsampled Even datasets. (**A**) Total assembled size of draft assemblies with respect to the total size of the reference metagenome; (**B**) fraction of the reference metagenome covered by the draft assembly, calculated by two different methods: metaQUAST (top panel) and minimap2 + BBTools (bottom panel).

Overall, long-read assemblers resulted in a total assembly size closer to the theoretical size, and also recovered a larger metagenome fraction, with some exceptions (Fig. 1). Nevertheless, large differences were detected for both metrics among the assemblers. All the assemblers were far from recovering the totality of the metagenome, both in the 3 Gbp and the 6 Gbp datasets (Fig. 1A). It must be noted that metaQUAST and minimap2 results were consistent for the long-read assemblers, but not for the short-read assemblers, where minimap2 metric was significantly higher (Fig. 1B). MetaFlye (both versions) yielded the best assemblies in terms of total metagenome size and metagenome recovery except for the minimap2 metric, followed by Pomoxis, Canu and Raven (previously known as Ra). Interestingly, assembly pipelines based on the miniasm algorithm (Pomoxis, Unycicler, and miniasm itself) presented huge variations in their performance. Unicycler and miniasm performed relatively well for the 3 Gbp dataset, but when using 6 Gb, the final assembly did not improve significantly in the case of miniasm, and the general performance was highly reduced for Unicycler. This is in contrast to Pomoxis, which produced the second most complete assemblies with both dataset sizes. Although based on miniasm, it is worth highlighting that Unicycler’s pipeline is designed for single isolate assembly, so reduced performance was expected for metagenomic studies. Finally, Redbean (previously known as wtdbg2) and Shasta resulted in poor assembly performance in comparison to the other long-read tools.

**Figure 2:**
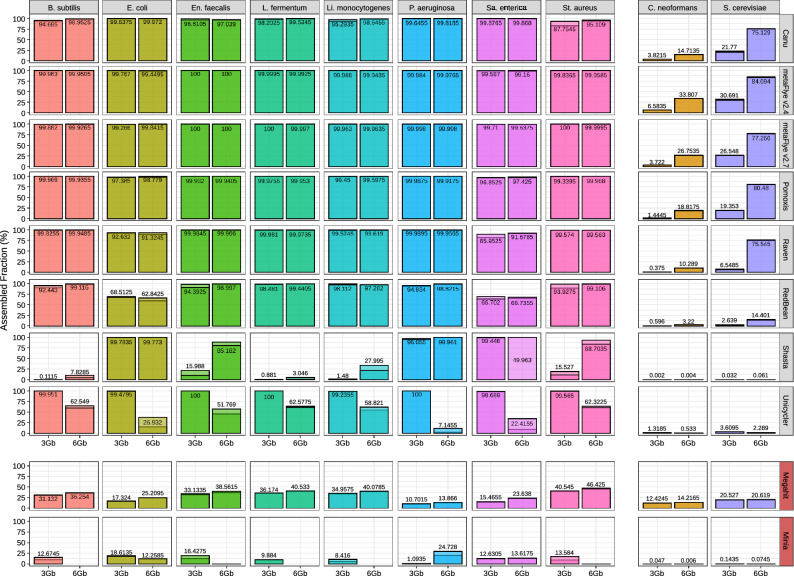
Fraction of the genome covered by the draft assemblies obtained using each tool, and for each individual microorganism (subsampled Even datasets). Miniasm assemblies are not shown, since it was not possible to evaluate them with metaQUAST.

MetaQUAST was used for further evaluating the degree of completeness of each individual draft genome (Fig. 2). As expected, yeast genomes were generally less recovered than bacterial ones, due to their lower abundance (2%) and higher size, explaining the low metagenome fraction generally recovered by all the assemblers (Fig. 1). In fact, the maximum average recovery fraction for the bacterial genomes was 99.92% (Supplementary Fig. S1). Minia and Megahit were not able to recover any single genome with high completeness (> 95% of genome coverage) in any dataset. For the 3 Gbp dataset, metaFlye (both versions) and Unicycler recovered the eight bacterial genomes with a high completeness level (> 98.6%), while Pomoxis achieved lower recovery fractions for two genomes (~ 96.9 to 97.4%). Raven and Canu resulted in reduced recovery percentages, but still retrieved all the prokaryotic genomes with a mean covered fraction greater than 85% and 87%, respectively. Redbean and Shasta achieved particularly low fractions of genome recovery.

For the 6 Gbp dataset, Unicycler performance decreased substantially as noted in Fig. 1, while Canu, Pomoxis, Raven and metaFlye achieved similar or better results. In general, metaFlye displayed the best performance on both dataset sizes in terms of genome recovery, closely followed by Pomoxis. This trend was also observed when analyzing the proportion of yeast genomes recovered by each tool. In this context, it is important to highlight that metaFlye’s ability to recover eukaryotic genomes was reduced when using metaFlye v2.7. This is due to the lower number of missassemblies retrieved by this metaFlye version, indicating that the reduced fraction of genome recovery is compensated with more reliable assemblies (Supplementary Fig. S2).

These results were confirmed when analyzing the Log mock community (Supplementary Fig. S3). Canu, metaFlye, Raven and Pomoxis were able to recover *Listeria monocytogenes* and *Pseudomonas aeruginosa* genomes (89.1% and 8.9% of total genomic DNA in the Log mock community, respectively) with a level of completeness higher than 99%. These assemblers also recovered a significant fraction of *Bacillus subtilis* (0.89% of total genomic DNA in the Log mock community). In fact, Raven was able to reconstruct > 99% of its genome using the 6 Gbp datasets, whereas metaFlye recovered ~ 98%. In this case, both tools outperformed Canu. Nevertheless, Raven did not recover a significant fraction of *Saccharomyces cerevisiae*, whereas Canu and metaFlye did (> 8%). Pomoxis worked correctly when using the 3 Gbp datasets, but failed to run with both 6 Gbp files. The other tools based on the miniasm algorithm also failed to run the 3 Gbp (Unicycler) and/or 6 Gbp datasets (miniasm). In all cases, the error was related to memory usage and accession (segmentation violation), and could not be solved. Nevertheless, using a computer with more RAM would help to easily overcome this problem. Shasta, RedBean, Minia and Megahit performed poorly in comparison to the other tools (Supplementary Fig. S3). It has to be noted that Shasta and RedBean were not originally designed to work with metagenomic data, which could result in problems to handle uneven coverages.

**Figure 3:**
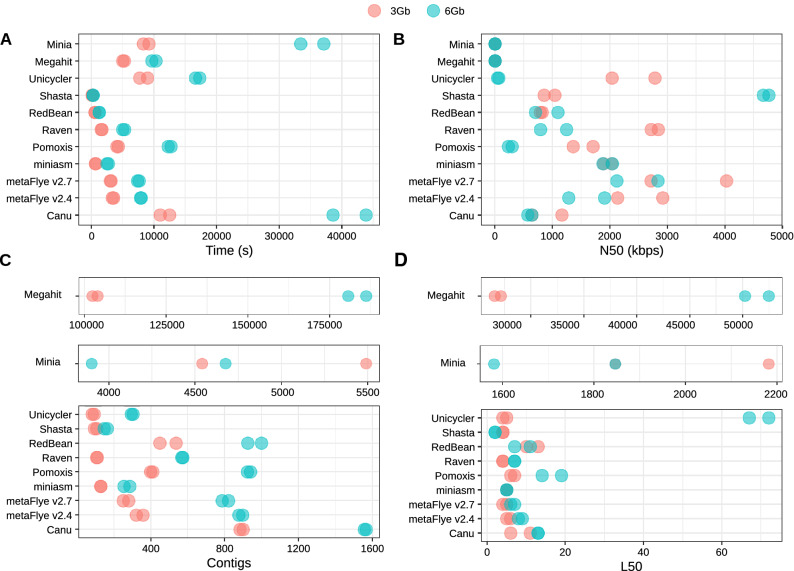
General assembly performance of each tool for the subsampled Even datasets. (**A**) Run time; (**B**) N50; (**C**) number of contigs; (**D**) L50.

Regarding the time consumed by each tool, Shasta was the fastest assembler (Fig. 3A). This tool was able to assemble the 6 Gbp datasets in only 285 s, approximately. RedBean and miniasm were the second and third most fast software, followed by Raven (1.5–1.9 times faster than metaFlye v2.7). MetaFlye was 1.4–1.7 times faster than Pomoxis, and 3.8–5.5 times faster than Canu, which proved to be the slowest tool. These trends were also found in the Log mock community (Supplementary Fig. S4), where Canu spent up to 22 h reconstructing a draft metagenome assembly from the 6 Gbp datasets. In this case, Raven was faster than metaFlye v2.7 for the 3 Gbp datasets, but not for the 6 Gbp ones.

General metagenome statistics (N50, L50, and number of contigs) were evaluated using QUAST (Fig. 3; Supplementary Table S3). It has to be stressed that the comparisons based on these metrics are difficult to analyze due to the large variation in the general performance among the different assemblers. For instance, Shasta resulted in the highest N50 and the lowest L50 values for the 6 Gbp dataset, but this tool was able to cover less than 35% of the metagenome. In fact, the total assembly size for Shasta was approximately 18–21 Mbp, in comparison to the 49–53 Mbp assembled by metaFlye.

As expected, short-read assemblers did not perform well with nanopore data, resulting in thousands (Minia), or even hundreds of thousands of contigs (Megahit). Interestingly, long-read assemblers resulted in more fragmented draft genomes when using the 6 Gbp datasets. Except for Shasta, the other long-read assemblers also reduced their N50 and increased their L50 and number of contigs score when using 6 Gbp. Goldstein et al.^9^ demonstrated that Canu assemblies improved with higher coverage when assembling bacterial isolates. This fact suggests that the loss of contiguity detected may be a direct consequence of a higher recovery rate of yeast genomes, which might be more fragmented. Indeed, assembly statistics of the Canu draft assemblies remained almost the same for the bacterial species when using 3 or 6 Gbp (Supplementary Table S4). Finally, metaFlye and Raven resulted in a more contiguous assembly with higher N50 and lower L50 in comparison to the other best performing tools (Canu and Pomoxis), for both 3 and 6 Gbp datasets (Fig. 3; Supplementary Table S3). Remarkably, metaFlye v2.7 yielded slightly better results than metaFlye v2.4 (Fig. 3B–D), and required less time (Fig. 3A).

**Figure 4:**
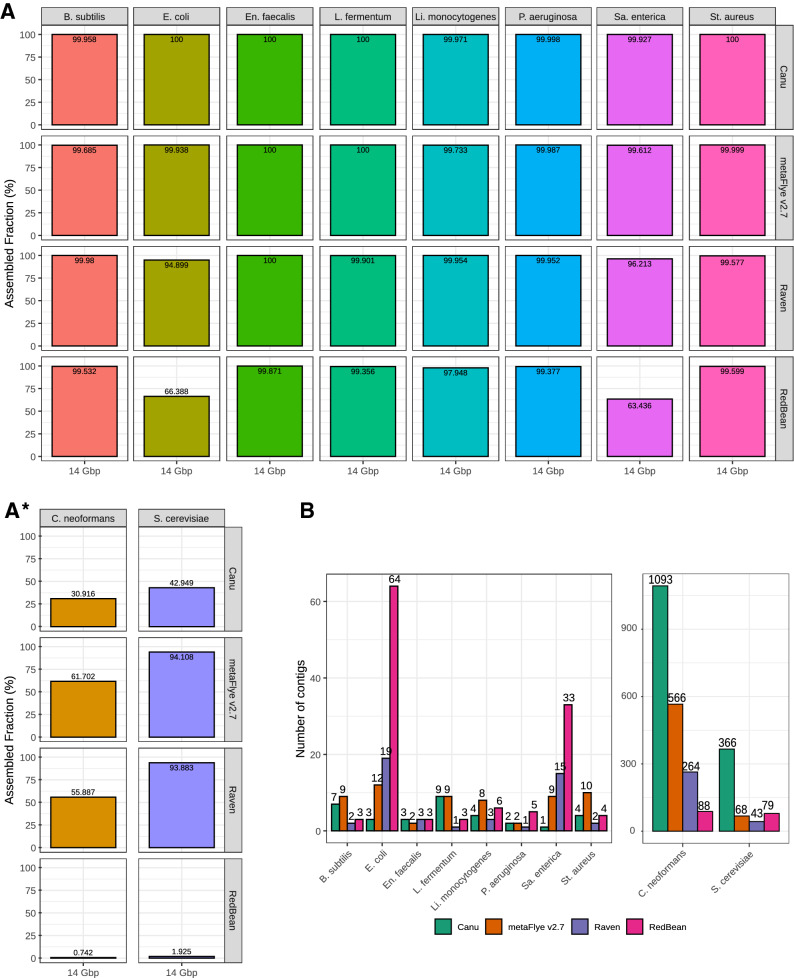
Even GridION (14 Gbp) assembly evaluation for the best performing tools. (**A**) Fraction of the genome covered by draft assemblies; (**B**) number of contigs for each microorganism.

ONT hardware, protocols and software are in constant development, leading to large improvements in short periods of time. Recently, an optimized DNA extraction and purification methodology has allowed to reach an average yield of ~ 15.9 Gbp per flowcell^46^. For that reason, we decided to run the most promising assemblers directly on GridION’s original data (Even mock community; 14 Gbp). RedBean was included because of its computational efficiency, which is a key factor for the analysis of deeply sequenced microbiomes. Results were similar to those obtained for the 3 and 6 Gbp (Fig. 4). Canu recovered the highest proportion of bacterial genomes, closely followed by metaFlye. Raven, once again, displayed problems when reconstructing the whole *Escherichia coli* and *Salmonella enterica* genomes, an issue also detected for RedBean in a more notable way. MetaFlye and Raven achieved a better recovery ratio than Canu for the yeast genomes. Overall, metaFlye genomes were more complete but less contiguous than the Raven draft assemblies, which presented a lower number of contigs for all the species with the exception of *E. coli* and *S. enterica* (Fig. 4B). This trend was also observed for the Log datasets (Supplementary Fig. S4). Remarkably, Raven was able to assemble two bacterial genomes in only one contig (*Lactobacillus fermentum* and *P. aeruginosa*), and retrieved four additional genomes in only 2–3 contigs. Finally, it was not possible to run Pomoxis on this dataset because of the unsolvable error previously described.

### Assembly accuracy

**Figure 5:**
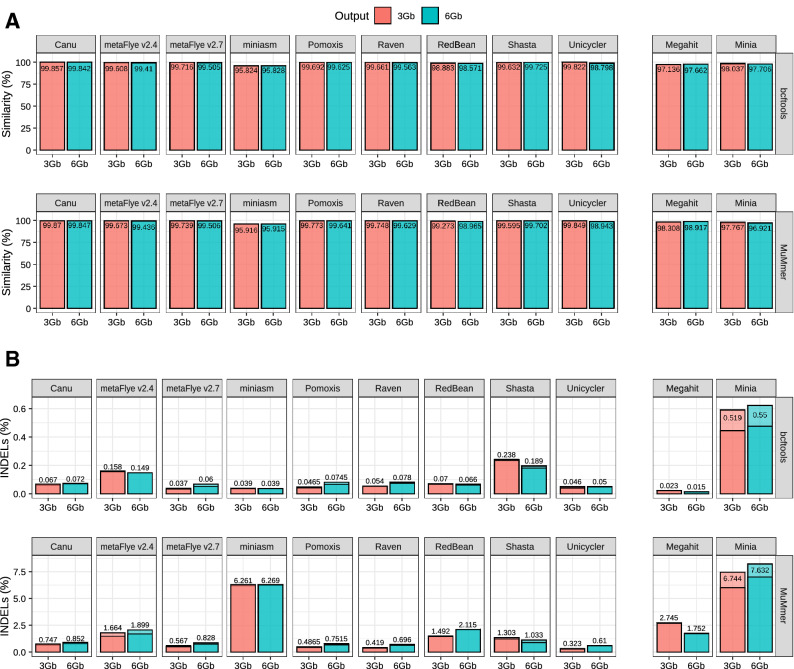
Assembly accuracy for the draft assemblies (subsampled Even datasets). (**A**) Percentage of similarity calculated as the total number of matches normalized by the metagenome size; (**B**) percentage of indels calculated as the total number of indels normalized by the metagenome size. In both cases, two different strategies were used: (top panel) alignment with minimap and evaluation with bcftools + ‘indels_and_snps.py’ in-house script; (bottom panel) alignment with MuMMer and evaluation with ‘count_SNPS_indels.pl’ script from Goldstein et al.^9^.

Sequencing errors are the biggest drawback of third generation sequencing platforms. These errors can reach the final assemblies, resulting in lower quality draft genomes. In order to evaluate how the different assemblers handle the specific error profile of ONT platforms, we analysed the total number of SNPs and indels present in each draft metagenome. As described in Methods, two different and complementary strategies were used to quantify these types of errors: (1) minimap2 + bcftools, and (2) MuMmer (Fig. 5). Both strategies relied on the alignment of the draft assemblies to the reference metagenome, composed by a mix of all the complete genomes of each strain present in the mock community.

Results were not fully consistent between the two methodologies, especially for the indels estimation, but they still showed similar trends. All the long-read assemblers retrieved draft metagenomes with an average similarity higher than ~ 98.9%, with the exception of miniasm, which resulted in an approximate accuracy of only 96%. This low accuracy could explain the inability of metaQUAST to evaluate miniasm assemblies. It has to be noted that the other pipelines based on miniasm, Pomoxis and Unicycler, incorporated several rounds of polishing via Racon^45^, which substantially reduced the number of SNPs and indels in the final draft assembly (see below).

Canu displayed a higher percentage of similarity for both methodologies and datasets, followed by Unicycler for the 3 Gbp dataset, and Shasta for the 6 Gbp one. Pomoxis, metaFlye, and Raven presented similarities over 99.5%. In the case of the indel profile, Unicycler and metaFlye v2.7 clearly outperformed Canu. Raven and Pomoxis also achieved a better indel ratio than Canu, except for the 6 Gbp dataset and the bcftools metric. Redbean, miniasm, and Shasta results were inconsistent between the two methodologies tested (Fig. 5).

### Biosynthetic gene cluster prediction

**Figure 6:**
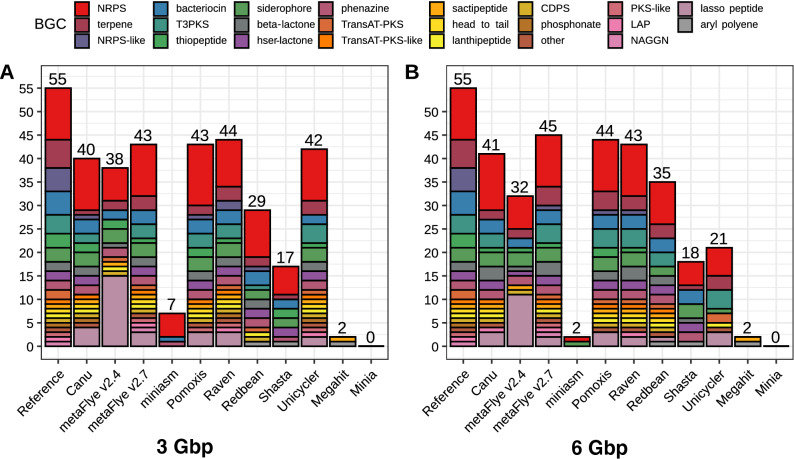
Number of biosynthetic gene clusters (BGCs) predicted by antiSMASH for each draft assembly in the Even GridION datasets. (**A**) BGCs predicted using the 3 Gbp dataset; (**B**) BGCs predicted using the 6 Gbp dataset.

Gene prediction is highly affected by genome contiguity, completeness and accuracy. BGCs are especially influenced by these factors, since they are usually found in repetitive regions which are often poorly assembled. AntiSMASH was used to assess the number of clusters found in the draft assemblies retrieved by each tool in comparison to the reference metagenome with the aim of evaluating BGC prediction on nanopore-based metagenomic assemblies (Fig. 6). As expected, none of the tools recovered the entire BCG profile, since metagenomes were not completely reconstructed (Fig. 1). Using the entire GridION dataset (14 Gbp) did not improve the number of BCGs recovered (Supplementary Table S5). Overall, when considering the total number of BGCs predicted and the similarity of the obtained profile compared to the reference profile, Raven displayed the best performance for both 3 Gbp datasets, whereas metaFlye v2.7 displayed the best performance for the 6 Gbp datasets. Pomoxis also achieved good predictions, outperforming Canu. All the predicted profiles presented an enrichment in lasso peptides (ribosomally-synthesized short peptides), which were not present in the reference profile. To further study this phenomenon, lasso peptides predicted by the different tools were searched using BLAST against the BGCs predicted in the reference metagenome. No hits were found, suggesting that these results might be prediction artifacts mainly caused by indels, which are probably introducing frameshift errors, and artificially increasing the number of short peptides being predicted (i.e. lasso peptides). In fact, metaFlye v2.7, which had a significantly lower indel ratio, retrieved fewer lasso peptides than metaFlye 2.4 (Fig. 5). We also corrected Pomoxis assemblies with Medaka, leading to a lower indel ratio (see the following section). Lasso peptides were not detected in Pomoxis + Medaka assemblies, highlighting the importance of indel correction for functional prediction (Supplementary Fig. S5).

### Polishing evaluation

Polishing is the process of correcting assemblies in order to generate improved consensus sequences. Input for polishing nanopore-based assemblies can be raw ONT reads (i.e. Racon or Medaka)^45^, raw electric signal (i.e. Nanopolish) (https://github.com/jts/nanopolish), or even high quality short reads (i.e. Racon)^45^. The state-of-art polishing workflow for nanopore sequencing consists of correcting the draft assemblies through several rounds of Racon (typically 2–4), followed by a single Medaka step.

**Figure 7:**
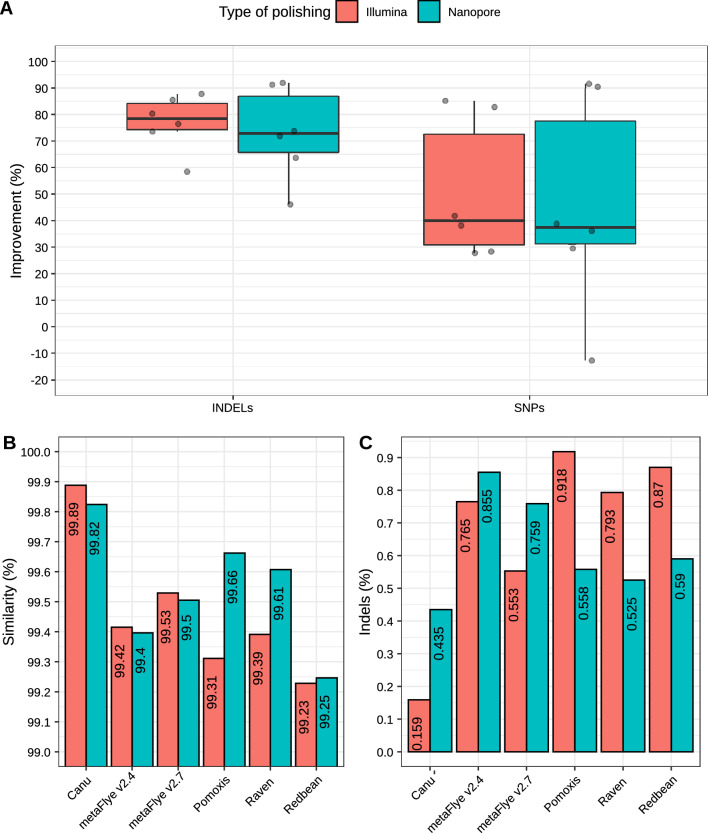
Polishing evaluation. (**A**) Percentage of improvement within the whole metagenome, taking as a reference the number of errors prior to polishing; (**B**) highest similarity percentage achieved by each tool; (**C**) best indel ratio achieved by each tool. Note that a different number of polishing rounds may be needed for achieving the highest similarity and the lowest indel ratio depending on the tool.

Some of the tested tools automatically incorporated Racon (Raven, Pomoxis and Unicylcer) in their pipelines, whereas the others included different algorithms for correcting the reads before (Canu) or after (metaFlye and ReadBean) the assembly process. Thus, we wanted to assess how various steps of polishing could affect the SNP and indel ratio of the different assemblers. Results were highly heterogenous (Fig. 7; Supplementary Table S6). Pomoxis and Raven drastically improved their accuracy after several rounds of polishing with the original ONT reads (Supplementary Table S6). In fact, accuracy with no polishing steps was close to 96%, as reported for miniasm (Fig. 5). Higher similarity percentages were observed after one round of Racon (1R) for Raven, and four rounds of Racon + one round of Medaka (4R + m) for Pomoxis. Redbean and metaFlye -which were run again without using their built-in polishers- also improved their accuracy after 1R or 4R + m, respectively. Canu presented a lower percentage of SNPs when no polishing steps were added to the pipeline (Supplementary Table S6). Nevertheless, all the tools drastically improved their indel ratio after 4R + m. The percentage of improvement varied between 41% (Canu) and 91% (Raven and Pomoxis) (Fig. 7A). It has to be highlighted that the lowest number of SNPs and indels was achieved by Canu, which is the only tool that carries out error correction before assembling the reads.

The error profiles were evaluated again to further assess whether polishing draft assemblies with high quality short reads led to improved assemblies. Albeit yielding heterogeneous results, all the tools achieved better indel ratios after four rounds of Racon correction with Illumina reads (Supplementary Table S6). In this case, all the assemblers improved their accuracy (% of similarity) after one (Canu and metaFlye) or four (Pomoxis, Raven and RedBean) Racon rounds. When comparing the highest scores obtained with Illumina-based correction to the highest scores achieved after ONT-based polishing (Fig. 7), the percentage of similarity was higher for metaFlye and Canu assemblies corrected with Illumina reads, and lower for Pomoxis, Raven and RedBean, where ONT polishing outperformed Illumina’s. A similar trend was observed for the indel ratio. This time, Illumina correction clearly enhanced the indel correction for metaFlye and Canu. In fact, Canu + Illumina correction retrieved the lowest indel ratio. Pomoxis, Raven and RedBean achieved a better indel correction with ONT reads.

## Discussion

Assembling shotgun sequencing data is often a key factor for characterizing the functional and taxonomic diversity of microbial communities. In the recent years, nanopore-based sequencers (Oxford Nanopore Technologies; ONT) are rapidly growing in popularity due to four basic reasons: (1) low cost, (2) long-read generation, (3) portability, and (4) real-time analysis. Several bioinformatic tools have been developed to handle nanopore sequences during the assembly process. Nevertheless, there is a lack of systematic, up-to-date, independent studies comparing the performance of the currently available tools. This work is aimed at filling this gap using the data previously published by Nicholls et al.^15^, which consisted of the ultra-deep sequencing of two different mock communities (Table 1) on GridION and PromethION platforms (ONT). These platforms follow the same sequencing principles as MinION, but they have a significantly higher output. For that reason, the datasets were subsampled in order to adapt their output to the current yield offered by MinION (3–6 Gbp)^9,34–39^, then extending the study to higher yields comparable with other recent works^46^.

Despite the relatively low complexity of the mock communities analyzed in this evaluation study, our results show that there is a huge variation in assembly results depending on the software chosen to perform the analysis. Minia and Megahit poorly reconstructed the microbial genomes (Figs. 1, 2) and produced highly fragmented draft assemblies (Fig. 3). This output was expected, since these assemblers are highly optimized to work on short reads, which are very different to the data generated by ONT sequencers.

Long-read assemblers (Canu, metaFlye, Unicycler, miniasm, Raven, Shasta and Readbean) also presented significant differences in the general assembly performance. This was expected too, since some of the tools were not specifically designed for assembling metagenomes (Supplementary Table S1). Overall, only metaFlye, Raven, and Canu worked well on all the tested datasets. They were able to recover the eight bacterial genomes from the Even dataset with a high degree of completeness, and also reconstructed a significant fraction of the yeast genomes. Draft assemblies were highly contiguous when using these three tools, as they were able to reconstruct bacterial genomes in only 1–19 contigs (Fig. 4B). Unicycler and, especially, Pomoxis, also performed well for some datasets and metrics, but failed to run in some cases (Supplementary Table S1). Both tools are pipelines based on miniasm that include further polishing steps by Racon. Miniasm alone was also unable to assemble the Log 6 Gbp dataset, indicating a lack of consistency of the algorithm for different microbial community structures. Finally, Shasta and RedBean (wtdgb2) retrieved incomplete assemblies and they did not provide any additional advantage other than computational efficiency.

Our results are in accordance with previous studies. MetaFlye has proved to outperform other tools in terms of metagenome recovery when using different mock communities^32,33^, although it must be noted that these previous studies did not include all the tools selected in the present benchmark. Canu also performed well in other studies^33^, and has been proposed for increasing the contiguity of metagenome assembled genomes recovered from real samples^46^. Nevertheless, its high computational cost limits the use of Canu for bigger datasets (Fig. 3, Supplementary Fig. S4)^33,46^. RedBean displayed a reduced performance in comparison to other long-read assemblers^32,33,46,47^. To the best of our knowledge, no other metagenome assembly benchmark has included Pomoxis, Shasta, or Raven. Wick and Holt^47^ evaluated different tools for single isolate assembly (not metagenomic assembly), and reported that Shasta was more likely to produce incomplete draft assemblies, while Raven was reliable for chromosome assembly, as also seen in our work. Although Pomoxis was not included in this last benchmark, another miniasm + Racon strategy was used. This strategy, that was reported to perform robustly among different genomic datasets, is equivalent to one of the pipelines used in the present study (here referred to as Unicycler). This observed robustness is in contrast to our results, supporting the idea that the intrinsic differential coverage of metagenomic datasets could be the cause of the inconsistency detected for miniasm in this benchmark.

Although sequencing errors are one of the main drawbacks of third generation sequencing platforms, the best performing tools (metaFlye v2.7, Canu, Raven and Pomoxis) achieved > 99.5% of accuracy in the final assemblies. Indels may be especially problematic, since they can introduce frameshift errors, which hinder functional prediction. After analyzing the different BGCs profiles, metaFlye and Raven demonstrated to reach better results and they outperformed Canu. This is in accordance with the indel ratio calculated for each tool (Fig. 5B). It has to be highlighted that these results were obtained by using ONT configurations explicitly recommended in the manual of each tool. The use of other tools (i.e. polishers) led to assemblies with enhanced quality. The lowest number of SNPs and indels were achieve after different rounds of polishing for some assemblers (Supplementary Table S6). As a consequence, the number of polishing rounds is variable and must be carefully chosen by the user. Correction with Illumina reads is a useful strategy for reducing the number of indels and SNPs produced by metaFlye and Canu, as also reported in Moss et al.^46^. The combination of Canu with polishing tools resulted in the best accuracy, especially when using Illumina reads for the correction.

Finally, time is a crucial parameter when choosing a bioinformatic tool, even more if considering MinION’s ability to generate real-time data. In this sense, metaFlye v2.7 was up to 6.7 times faster than Canu, which was the slowest tool tested on this benchmark. Raven was even faster than metaFlye, and tended to generate fewer contigs (Fig. 3, Supplementary Fig. S4; Supplementary Table S3).

Taken together, our results show that nanopore data (accommodated to current MinION’s output) can lead to highly contiguous and accurate assemblies when using the proper tools, with no need of complementary sequencing with Illumina. From all the tested software, metaFlye v2.7 resulted the best in terms of metagenome recovery fraction and total metagenome assembled size. Raven achieved slightly lower genome fractions than metaFlye, but was faster and generally retrieved a lower number of contigs. Canu was the most accurate tool and introduced fewer indels when combined with polishing tools, but its assembly process also demonstrated to be time consuming. Pomoxis and other miniasm-based pipelines are also promising, but their inconsistency problems should be addressed. This work may help software developers to design new bioinformatic tools optimized for nanopore-based shotgun metagenomic sequencing, although further research is still needed in order to benchmark the different assemblers on more complex microbial communities.

## Conclusions

Shotgun metagenomic sequencing based on short reads usually results in highly fragmented metagenomes, thus complicating downstream analyses such as the recovery of individual genomes or the prediction of complex and repetitive gene structures (i.e. biosynthetic gene clusters, CRISPR-CAS systems, etc.). This work demonstrates that, despite the high error intrinsic to third-generation sequencing platforms, nanopore data alone can overcome these limitations and retrieve extremely contiguous genomes directly from simple microbial communities. However, there is a huge variation in assembly performance depending on the chosen software. In general terms, metaFlye could be defined as the best suited assembler for nanopore metagenomic data. This tool leads to the highest metagenome recovery ratio and performs robustly among the tested datasets. Raven also performed well and required less time to perform the analyses. Canu is more suitable when lower error rates are required, but draft assemblies should be polished in order to reduce the number of indels. Polishing with short reads does not necessarily improve the quality of the draft assemblies but, in combination with Canu, it can lead to the most accurate metagenome reconstruction. Overall, this work demonstrates the suitability of using nanopore sequencing alone for assembling low-complexity microbial communities, and paves the way towards the standardization of bioinformatic pipelines for long-read sequencing data.

## Supplementary information

Supplementary Table 1

Supplementary Table 2

Supplementary Table 3

Supplementary Table 4

Supplementary Table 5

Supplementary Table 6

Supplementary Figure 1

Supplementary Figure 2

Supplementary Figure 3

Supplementary Figure 4

Supplementary Figure 5

Supplementary Legends

## Data Availability

Raw data was deposited in the NCBI database under the BioProject number PRJNA564477 (https://www.ncbi.nlm.nih.gov/bioproject/564477). Raw datasets from Nicholls et al.^15^ can be downloaded from the ENA (https://www.ebi.ac.uk/ena/data/view/PRJEB29504). All the code used in this study is publicly available at doi: https://doi.org/10.5281/zenodo.3935763. It includes the bash scripts designed for the automatic execution of the different bioinformatic analysis, the R code and CSV tables for figure construction, and other in-house and third-party scripts needed to reproduce the analyses.
