# Supplementary material for: Assembly methods for nanopore-based metagenomic sequencing: a comparative study
Source: Sci Rep. 2020 Aug 12;10:13588. doi: 10.1038/s41598-020-70491-3 (PMC7423617; doi:10.1038/s41598-020-70491-3)
Supplement: Supplementary file 1 — Supplementary Table 1 [file 41598_2020_70491_MOESM1_ESM.docx]

**Supplementary Table S1.** List of assemblers selected for the benchmark.

| **Assembler** | **Version** | **Type** | **Problems encountered** | **Intended scope** |
| --- | --- | --- | --- | --- |
| **MetaSPAdes** | v3.13.0 | Short-read | RAM memory error | Metagenomic assembly |
| **Megahit** | v1.1.4-2 | Short-read | No problems reported | Metagenomic assembly |
| **Minia** | v.2.0.7 | Short-read | No problems reported | Metagenomic assembly |
| **Canu** | v1.8 | Long-read | No problems reported | General usage |
| **HINGE** | --- | Long-read | Config files could not be properly modified | General usage |
| **Miniasm** | v0.3(r179) | Long-read | Failed to run with the 6 Gbp Log datasets | General usage |
| **MetaFlye** | v2.4 | Long-read | No problems reported | Metagenomic assembly |
| **MetaFlye** | v2.7 | Long-read | No problems reported | Metagenomic assembly |
| **Pomoxis** | v0.3.2 | Long-read | Failed with the 6 Gbp Log datasets and the 14 Gbp Even GridION dataset | General usage |
| **Raven** | v0.0.8 | Long-read | No problems reported | General usage |
| **Redbean** | Wtdbg v2.5 | Long-read | No problems reported | General usage |
| **Shasta** | v.0.4.0 | Long-read | No problems reported | General usage |
| **Unicycler** | v0.4.8-beta | Long-read | Failed with the 3 Gbp Log datasets | Isolated bacteria |
