# Supplementary material for: Assembly methods for nanopore-based metagenomic sequencing: a comparative study
Source: Sci Rep. 2020 Aug 12;10:13588. doi: 10.1038/s41598-020-70491-3 (PMC7423617; doi:10.1038/s41598-020-70491-3)
Supplement: Supplementary file 2 — Supplementary Table 2 [file 41598_2020_70491_MOESM2_ESM.docx]

**Supplementary Table S2.** Detailed list of the commands run for the different assemblers and polishers.

| **Assembly** | |
| --- | --- |
| **Software** | **Commands** |
| **Megahit** | **1.** megahit -t *number_threads* -r *dataset_trimmed.fastq* --out-prefix *prefix_output_files* -o *output_folder* |
| **Minia** | **1.** minia *dataset_trimmed.fastq* *kmer_size min_abundance estimated_genome_size prefix_output_files* |
| **Canu** | **1.** canu -d *output_folder* -p *prefix_output_files* genomeSize=*genome_size(number[g\|m\|k])* -nanopore-raw *dataset_trimmed.fastq* |
| **MetaFlye (v2.4 and v2.7)** | **1.** flye --nano-raw *dataset_trimmed.fastq* --out-dir *output_folder* --genome-size *genome_size(number[g\|m\|k])* --threads *number_threads* --meta --plasmids |
| **Miniasm** | **1.** minimap2 -x ava-ont -t *number_threads* *dataset_trimmed.fastq* dataset_trimmed.fastq \| gzip -1 > *[prefix_input_files].paf.gz* **2.** miniasm -f *dataset_trimmed.fastq [prefix_input_files].paf.gz* > *[prefix_input_files].gfa* **3.** awk '/^S/{print ">"$2"\n"$3}' *[prefix_input_files].gfa* \| fold > *[prefix_output_files].fa* |
| **Pomoxis** | **1.** source [Absolute_path_pomoxis_software_folder]/venv/bin/activate **2.** mini_assemble -i *dataset_trimmed.fastq* -o *output_folder* -p *prefix_output_files* -t *number_threads* |
| **Raven** | **1.** raven -t *number_threads* dataset_trimmed.fastq > [prefix_output_files].fa |
| **Redbean** | **1.** wtdbg2 -x ont -g *genome_size(number[g\|m\|k])* -t *number_threads* -i *dataset_trimmed.fastq* -fo *prefix_output_1_files* **2.** wtpoa-cns -t *number_threads* -i *[prefix_output_1_files].ctg.lay.gz* -fo *[prefix_output_2_files].ctg.fa* |
| **Shasta** | **1.** shasta-Linux-0.4.0 --threads *number_threads* --memoryBacking *arg([disk\|4K\|2M)* --memoryMode *arg([anonymous\|filesystem])* --input *dataset_trimmed.fastq* --assemblyDirectory *output_folder* |
| **Unicycler** | **1.** unicycler -l *dataset_trimmed.fastq* -t *number_threads* -o *output_folder* |
| **Polishing** | |
| **Software** | **Commands** |
| **Racon** | **1.** minimap2 -x map-ont -d *assembly_unpolished.mmi* *assembly_unpolihed.fasta* **2.** minimap2 -ax map-ont -t *number_threads* *assembly_unpolished.mmi reads_(ONT/Illumina).fastq* > *overlaps.sam* **3.** racon -t *number_threads reads_(ONT/Illumina).fastq overlaps.sam assembly_unpolished.fasta* > *assembly_polished.fasta* |
| **Medaka** | **1.** source activate medaka **2.** medaka_consensus -i *reads_ONT.fastq* -d *assembly_unpolished.fasta* -o *output_folder* -t *number_threads* |
