# Supplementary material for: Assembly methods for nanopore-based metagenomic sequencing: a comparative study
Source: Sci Rep. 2020 Aug 12;10:13588. doi: 10.1038/s41598-020-70491-3 (PMC7423617; doi:10.1038/s41598-020-70491-3)
Supplement: Supplementary file 4 — Supplementary Table 4 [file 41598_2020_70491_MOESM4_ESM.docx]

**Supplementary Table S4.** Canu’s basic assembly metrics for the subsampled GridION datasets.

|  | **3Gb** | | | **6Gb** | | |
| --- | --- | --- | --- | --- | --- | --- |
|  | **Contigs** | **N50 (bp)** | **L50** | **Contigs** | **N50 (bp)** | **L50** |
| ***Bacillus subtilis*** | 34 | 298,071 | 5 | 17 | 655,353 | 3 |
| ***Enterococcus faecalis*** | 13 | 388,478 | 3 | 10 | 747,976 | 3 |
| ***Escherichia coli*** | 5 | 2,669,962 | 1 | 6 | 4,941,166 | 1 |
| ***Lactobacillus fermentum*** | 13 | 402,806 | 2 | 14 | 4,941,166 | 1 |
| ***Listeria monocytogenes*** | 18 | 4,942,769 | 1 | 14 | 2,747,940 | 2 |
| ***Pseudomonas aeruginosa*** | 3 | 5,593,153 | 1 | 4 | 2,747,940 | 2 |
| ***Salmonella enterica*** | 3 | 4,942,769 | 1 | 11 | 2,075,612 | 2 |
| ***Staphylococcus aureus*** | 17 | 769,443 | 2 | 17 | 640,396 | 3 |
| ***Cryptococcus neoformans*** | 199 | 3,722 | 51 | 807 | 5,9140 | 189 |
| ***Saccharomyces cerevisiae*** | 596 | 6,062 | 142 | 684 | 18,390 | 145 |
