# Supplementary material for: Assembly methods for nanopore-based metagenomic sequencing: a comparative study
Source: Sci Rep. 2020 Aug 12;10:13588. doi: 10.1038/s41598-020-70491-3 (PMC7423617; doi:10.1038/s41598-020-70491-3)
Supplement: Supplementary file 12 — Supplementary Legends [file 41598_2020_70491_MOESM12_ESM.docx]

**Supplementary Figure S1.** Average recovery fraction for the bacterial genomes.

**Supplementary Figure S2.** Number of missassemblies detected in metaFlye v2.4 vs metaFlye v2.7.

**Supplementary Figure S3.** Fraction of the genome covered by the draft assemblies obtained with each tool, and for each individual microorganism (Log datasets). Minimap2 + miniasm assemblies are not shown, since it was not possible to evaluate them with metaQUAST. Only microorganisms with >1% genome fraction recovered for at least one long-read assembler are shown.

**Supplementary Figure S4.** General assembly performance of each tool for the subsampled Log datasets. (A) Run time; (B) N50; (C) Number of contigs; (D) L50.

**Supplementary Figure S5.** Number of biosynthetic gene clusters (BGCs) predicted by antiSMASH for Pomoxis and Pomoxis + one round of Medaka polishing.
